# Adjustment of Mechanical Properties of 3D Printed Continuous Carbon Fiber-Reinforced Thermoset Composites by Print Parameter Adjustments

**DOI:** 10.3390/polym16212996

**Published:** 2024-10-25

**Authors:** Md Atikur Rahman, Luke Gibbon, Md Zahirul Islam, Eric Hall, Chad A. Ulven

**Affiliations:** Mechanical Engineering Department, College of Engineering, North Dakota State University, Fargo, ND 58108, USA; mdatikur.rahman@ndsu.edu (M.A.R.); luke.r.gibbon@ndsu.edu (L.G.); mdzahirul.islam@ndsu.edu (M.Z.I.); eric.hall.1@ndsu.edu (E.H.)

**Keywords:** additive manufacturing, 3D printing, continuous fiber-reinforced composites, carbon fiber, photo curable resin, dual cured composites, composite mechanical property adjustment

## Abstract

Reinforcing thermoset polymers with continuous carbon fiber (CF) tow has emerged as a promising avenue to overcome the thermal and mechanical performance limitations of 3D printed polymeric structures for load-bearing applications. Unlike traditional methods, manufacturing continuous fiber-reinforced composites by 3D printing has the unique capability of locally varying the mechanical properties of the composites. In this study, continuous CF thermoset composite specimens were printed with varying line spacing, resin flow rate, and nozzle sizes. The resin flow rates for different line spacings and nozzle sizes were optimized by topographic analysis. Printed composite mechanical properties were evaluated, and their trends were correlated with the trend of print parameter changes. Results showed that tensile strength and modulus could be altered and improved by ~50% by adjusting the printing process parameters. Higher composite strength and modulus were obtained by shortening the line spacing and nozzle diameter.

## 1. Introduction

Additive manufacturing (AM), often referred to as 3D printing, has been around for a little over three decades [1]. However, it is only in the past decade that it has truly gained momentum as a revolutionary advancement in manufacturing technologies. AM now offers ways to create complex functional parts using metals, ceramics, thermoplastic, and thermoset materials [2,3,4,5]. While many AM techniques focus on printing with thermoplastics, such as fused deposition modeling (FDM) [6,7,8], thermosets are becoming increasingly popular in industrial settings due to their superior mechanical properties and heat resistance. Techniques like stereolithography (SLA) [9,10,11], digital light processing (DLP) [12,13], inkjet, and liquid deposition modeling (LDM) [1] are among the common methods used for 3D printing with thermosets.

Continuous fiber-reinforced thermoset composites are extensively utilized in high-performance composite applications, including aerospace, high-end automotive, marine, sporting goods, prosthetics, medical devices, and wind turbines [14,15,16,17,18]. These composites are often preferred over thermoplastic composites in such demanding environments because thermosets, with their lower initial viscosity, allow for greater fiber impregnation. This results in better strength, stiffness, and increased resistance to creep [19]. The growing interest in thermosetting composites stems from their potential to create higher-performance materials and their ability to be tailored to specific needs, representing a promising opportunity for AM in composite production [20,21,22]. However, challenges like low viscosity, difficulties of fiber handling, and snap curing have slowed down the commercial development of thermosetting composite 3D printers [23].

Due to the unique advantages of AM/3D printing manufacturing methods, the development of 3D printing technologies for fiber-reinforced polymer composites has gained massive interest in both academic and industrial settings. 3D printed fiber-reinforced thermoplastic composites have experienced better advancements compared to their thermosetting counterparts [24]. The Markforged company has successfully commercialized multiple 3D printers that are able to print high-quality continuous fiber-reinforced thermoplastic composites [25]. Research has also explored printing short and continuous fiber composites using fused filament fabrication (FFF) [26,27]. In this setup, a spool of filament, usually thermoplastic, is fed through a heated extruder along with a continuous strand of carbon fiber (CF) [28]. Techniques like nozzle impregnation [29,30,31] and pre-impregnation [6,32,33] of polymers for reinforcing continuous fibers in FFF printers have been studied. While effective, these methods have mainly been limited to thermoplastic filaments such as ABS. The mechanical properties of these 3D printed composites have been further improved by studying the print orientation [34] and numerical analysis [35]. However, significantly higher mechanical strengths were reported by the printed thermoset composites [36]. In addition to better mechanical properties, unlike thermoplastic composites, thermoset composites have higher molding efficiency due to their low viscosity. However, due to the cross-linked structure of thermoset polymers, these composites exhibit brittleness and lower impact toughness compared to the thermoplastic composites [37].

The development of 3D printing thermoset composites has been comparatively slower due to its unique challenges. Despite the progress made, several limitations remain in 3D printing thermoset composites. Generally, fiber-reinforced thermoset composite 3D printing falls into two categories: short fiber and continuous fiber [38,39,40]. Many existing techniques are confined to producing short fiber composites [41]. In these methods, thermoset resin is mixed with short fibers and then printed using conventional processes. SLA printing is one such method, where ultraviolet (UV) light is projected onto a flat surface of UV-curable resin. The UV light triggers polymerization, solidifying the resin. This is usually carried out in a “bottom-up” process, where the print bed rises from a vat of resin as each layer is completed. The process repeats to create layers of short fiber composite. While the fibers are not aligned, they still contribute to the final properties of the printed part. Although this approach can improve certain mechanical properties, significant performance enhancements are typically achieved only when the thermoset resin is combined with continuous dry fibers [32,39,41]. A comparative study was conducted for the performance of SLA 3D printed composites reinforced with particle, short, and woven fabric [42]. Despite demonstrating the best mechanical properties, the quality finish was missing in the fabric-reinforced composite prints in this study.

The first successful 3D printing of continuous CF-reinforced thermoset composites was achieved by Hao et al. using a direct ink write (DIW) process [39]. DIW techniques offer 3D printing of composites without requiring any light or heat; thus, DIW printing is less energy-intensive and more cost-effective [43]. However, DIW printing has its limitations, particularly in creating complex or tall structures [43]. Another approach, liquid deposition modeling (LDM), enables the printing of continuous fiber-reinforced thermoset composites with instant curing through violet light exposure [44]. In this process, a photocurable resin is pumped through a nozzle, where it mixes with dry fiber. As the mixture is deposited, a violet laser beam cures the resin just enough to hold the material in place, layer by layer. The final curing can be completed using a thermal initiator followed by post-cure heating in an oven [45]. Both LDM and DIW techniques can achieve better wet-out compared to their thermoplastic counterparts due to the low-viscosity resins utilized in DIW and LDM [46]. A nozzle mechanism was developed to push out continuous fiber by the sheer force of the resin flow for 3D printers [38]. Some studies have also looked into pre-impregnating the dry fiber with a liquid thermoset resin and partially curing it before feeding it through the printing nozzle [47]. This method has been used to optimize printing parameters and enhance the mechanical performance of 3D printed thermoset composites [48]. The Continuous Composites company has demonstrated a successful prototype 3D printer that is able to print continuous CF-reinforced thermoset composites [36].

3D printed continuous fiber-reinforced thermoset composites hold massive potential in manufacturing functional composites with tailor-crafted local properties [49,50]. Traditional manufacturing techniques have developed composite structures that have controlled deformation shape [51], predictable fracture, and viscoelastic behaviors [52,53]. Such composite structures have been utilized in aerospace [54], military [55], and competitive automotive [56] industries. While this field continues to evolve, despite having technical advantages, there remains a lack of extensive literature on 3D printing continuous fiber-reinforced composites with locally adjusted and tailor-crafted physical properties [57]. The current composite AM technologies require additional robotic assistance as part of the process of manufacturing large composite structures. A few of these robotic assistants are required for handling the materials, postprocessing, and assembly of parts of the composite structures. The stiffness and deformation characteristics of the printed composite parts are significant while assembling them in a robot-assisted process, and these mechanical characteristics require careful engineering for a consistent robot-assisted process.

Local alteration of composite material properties without changing the design geometry is potentially a very useful tool to engineer composite parts for robot-assisted manufacturing and assembly processes. The adjustment of properties could be carried out by the adjustment of several process parameters. Effects of print speed, compaction pressure, and layer thickness have been studied for heat-extruded 3D printed thermoset composites [58]. The study reported decreased tensile strength for increased compaction force and optimized settings for print speed and layer thickness. The limitations of the cornering radius of the printing path have been studied for a shear flow extrusion-based 3D printing technique for thermoset composites [38]. The effect of spacing and vacuum was optimized for maximum mechanical properties [59]. The effects of process parameters were also studied by utilizing numerical simulation methods [60]. These studies reported a non-linear response to the mechanical properties of 3D printed thermoset composites with varying print parameters.

This study presents a framework for altering the mechanical properties of 3D printed thermoset composites by adjusting process parameters. By utilizing continuous CF tow and a dual-curable (UV and thermal) liquid thermoset resin system, this study investigated the 3D printed composites’ mechanical behavior with altered fiber placement and material delivery rate. By changing these process parameters, composites with varying compositions were 3D printed. An optical topographic analysis method was utilized to optimize the parameter adjustment. Changes in the compositions and mechanical properties of the printed composites were correlated with the parametric adjustments.

## 2. Materials and Methods

### 2.1. Materials

To attain the sharpest print resolution, the CF tow count was chosen to be 1K. This 1K CF was graciously provided by Toray (Toray, Type T300, Filaments 1000-40A, TorayCMA, Tacoma, WA, USA) [61]. A total of 1k CF tow was chosen for the finest print resolution and easier resin impregnation.

A UV-curable resin from Peopoly (type: deft white, Peopoly, Los Angeles, CA, USA) was used as the matrix material [62]. This is an acrylic-based resin system cured at the exposure of 405 nm wavelength light. This is a low-viscosity liquid resin system that facilitates good impregnation in the fiber tow. This resin also boasts a large curing depth and low exposure requirement. Thermal initiator Luperox P (tert-butyl peroxybenzoate 98%, Sigma-Aldrich, Saint Louis, MO, USA) was added at 2% by weight to the Peopoly resin for proper matrix curing with additional heating [63]. This thermal initiator was chosen for its low viscosity and good miscibility with the acrylate resin system [44,64]. The curing performance of the initiator was analyzed by DSC tests of the composites (Section 2 and Section 3). The resin and thermal initiator were mixed for 5 min at 2500 rpm using a SpeedMixer (Hauschild Engineering, Model: SpeedMixer, Type: DAC-150 FVZ, Hamm, Germany). Batches of 50 g resin were mixed at a time, and then the mixtures were degassed at −85 kPa using a vacuum chamber (VWR, Sheldon Manufacturing Inc., Model: 1415M, Cornelius, OR, USA). Mechanical properties of composites constituents’ are shown in Table 1.

### 2.2. Fiber Tow Prepregging

**Figure 1:**
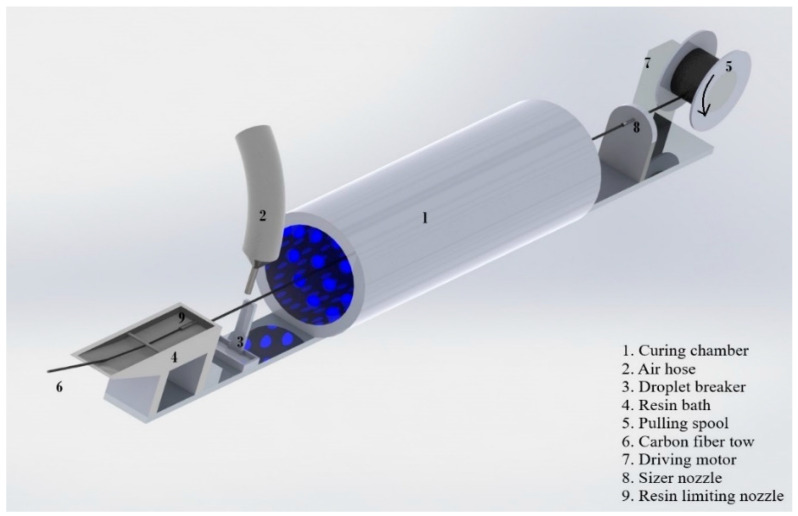
Schematics of prepreg production [19].

A short (2 mm) mixing chamber, inside the dispensing nozzle, was utilized for impregnating and encapsulating the CF tow with liquid thermoset resin. This short length for encapsulating the CF tow was selected to reduce the friction force experienced by the delicate CF tow while moving through the printing nozzle. This friction force and the concentrated friction force at the nozzle tip could damage and fray the CF tow while being laid on the print surface. On the contrary, a longer mixing chamber can facilitate better resin impregnation in the tow. Fiber prepregging equipment was constructed and utilized to pre-impregnate the fiber tow. Figure 1 is a schematic representation of this equipment. In this prepregging equipment, fiber tow was passed through a liquid resin bath, where the resin impregnated the fiber. The impregnated CF tow exited the bath through a 0.60 mm orifice. After exiting the orifice, a scrapper and an air blower removed any resin droplet formation around the outer surface of the tow. This ensured a consistent prepreg tow diameter. After this, the pre-impregnated CF tow was passed through a curing chamber. The curing chamber was a (160 mm long × 42 mm diameter) cylindrical chamber coaxial to the fiber tow axis. Inside the chamber, an array of 405 nm wavelength LEDs illuminated the outer surface of the tow and partially cured the liquid thermoset resin in the peripheral region of the tow. This partial curing made the fiber tow fraying resistant against the friction forces while printing.

**Figure 2:**
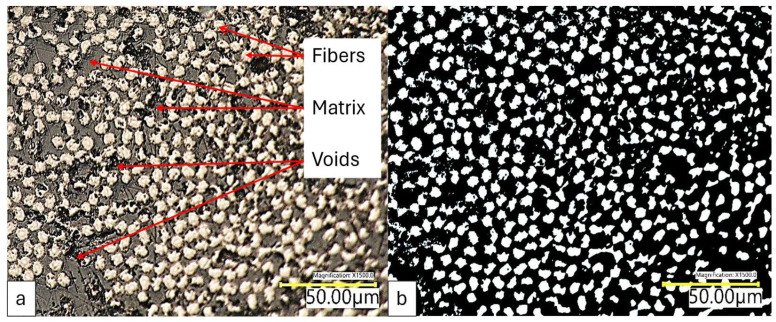
(**a**) Microscopic image of prepreg cross-section, (**b**) Monochromatic image of microscopic cross-section of prepreg.

Besides making the CF tow prepreg fray-resistant, the prepregging also facilitated good resin impregnation within the tow. This impregnation performance is critical for an efficient load transfer between the fiber filaments and the matrix. Microscopic inspection of the cross-section of tow prepreg revealed a satisfactory resin impregnation within the tow. Figure 2 shows a microscopic image of a cross-section of the prepreg tow. The microscopic image shows minimal void within the prepreg.

### 2.3. 3D Printing Equipment

**Figure 3:**
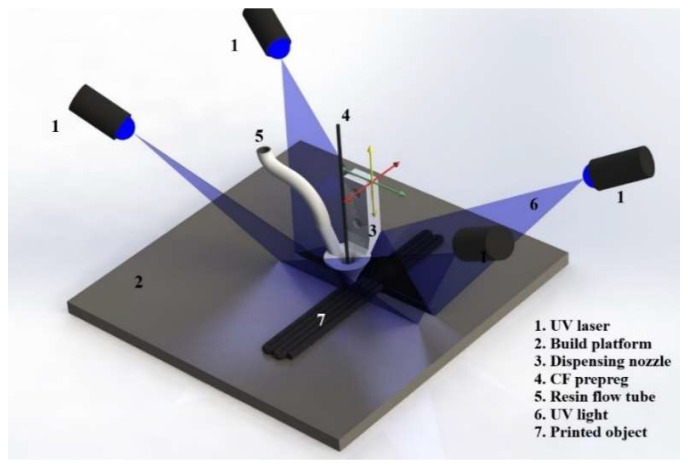
Schematic of 3D printing components [19].

A 3D printing system was constructed using the X and Y movement arms from a repurposed gantry system. The Z movement of the nozzle was placed at the XY scanning arm. The fiber and resin feeding system was also placed in the printing arm. Figure 3 is a 3D visualization of the printer component arrangement. A series of 4 lasers (Jolooyo, Wuhan, China, 405 nm wavelength, 150 mW output) illuminated and cured the liquid resin around the prepreg. The lasers were focused on a line shape, and their illumination sequence changed depending on the print path direction.

The prepreg tow was pulled through the printing nozzle by the tension from the print. Pre-calculated resin flow rate (${\dot{V}}_{r}$) to the nozzle was maintained during the printing process. The purpose of the resin pump was to provide the required amount of resin to the printing nozzle. As the print head moved, prepreg was pulled through the nozzle by the tension generated from the solidified points of the printed object. This draw of fiber prepreg carried the resin, which was delivered to the nozzle by the resin pump, through the exit tip. The reflected laser light from the print surface could create unwanted resin curing at the nozzle tip. This could cause build-up at the resin tip and thus change the tip geometry. This could result in nozzle-print surface interference, nozzle clogging, and fiber snapping. To avoid these situations, the laser beam was focused at a 4 mm offset from the nozzle tip. This is a tradeoff between process stability and cornering accuracy, which is a design consideration while generating G-codes for the printing of composite objects.

**Figure 4:**
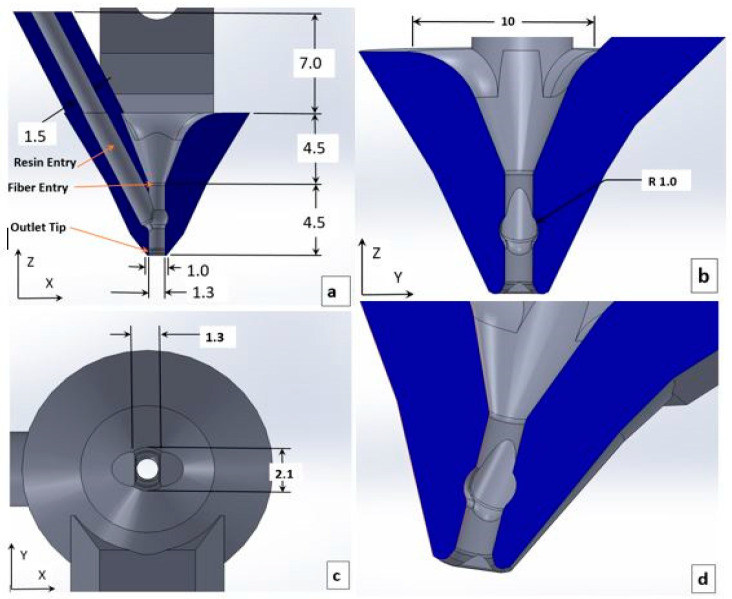
(**a**) Cross-section of the printing nozzle in XZ plane. (**b**) Cross-section of the printing nozzle in YZ plane. (**c**) Bottom view of the nozzle tip. (**d**) 3D view of the cross-section in XZ plane. The dimensions are in mm [19].

The geometry and size of the nozzle were crucial aspects of the design. The nozzles of this composite 3D printer were 3D printed using a Formlabs 3D printer (Model: Form 2, Somerville, MA, USA). As shown in Figure 4, the nozzle system had two inlets, one for resin and the other for prepreg. 3D printed nozzles, with rounded edges at the exit tip, were used to maintain a smooth flow of resin and prepreg. The inner diameter of the nozzle $\left(D_{n}\right)$, spacing between print lines ($l_{s}$) and resin flow rate (${\dot{V}}_{r}$) were varied, and the print performance of the varied parameters was evaluated.

**Figure 5:**
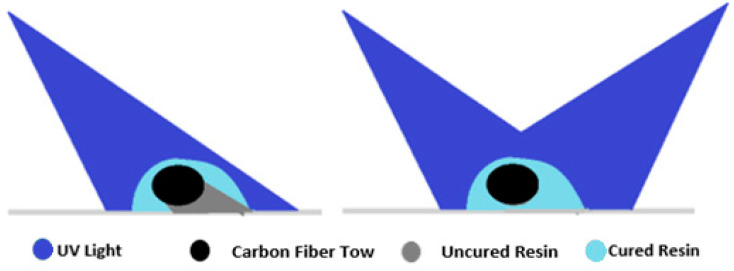
Laser orientation and shadow effect.

Due to the opacity of CF and its shadow effect, the exposure of violet laser light does not achieve a full cure. The shadow effect is demonstrated in Figure 5. To overcome the shadow effect, a bidirectional laser beam and dual-curable resin were utilized for 3D printing the composites. As shown in Figure 5, two laser beams minimized the shadow underneath the fiber tow and maximized the degree of cure in the laser curing stage. After completion of the printing process, the printed parts were post-cured at 130 °C for 1 h 30 min in a VWR oven (VWR, Sheldon Manufacturing Inc., Model 1350FM-2, Cornelius, OR, USA). A thermal initiator and post-curing process were added to enhance curing. The thermal initiator generated reactive species under the elevated temperature and cross-linked the remaining uncured resin after the laser curing stage. Luperox P was mixed into resin as the thermal initiator (tert-butyl peroxybenzoate 98%) to facilitate this thermal curing. The dual curing functionality ensured full cure of the matrix and facilitated efficient load transfer between the fiber and the matrix. It is also worth noting that upon generating the free radicals by the thermal initiator, a small amount of void could be produced in the matrix, which can marginally reduce the mechanical performance of the composites. This was a design consideration while adjusting the mixing ratio and resin flow rates.

### 2.4. Parameter Selection

**Figure 6:**
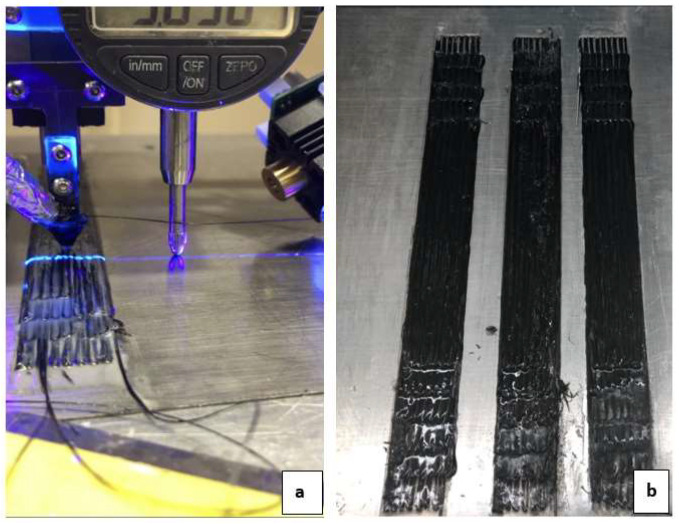
(**a**) Composite specimens being printed. (**b**) Composite rectangular bars.

To determine the tightest packing achievable with this 3D printer, a qualitative and quantitative study was needed. A large nozzle diameter ($D_{n}=$ 1.2 mm) and a wide line spacing ($l_{s}=$ 1.2 mm) were selected for the initial configuration. Printed samples with this configuration are shown in Figure 6. These values were selected based on a previous study [47]. The line spacing ($l_{s}$) is gradually reduced from this initial configuration until the setup fails to print. Then, the same iteration process was conducted with $D_{n}=$ 1 and $D_{n}=$ 0.8 mm nozzles. The $D_{n}=$ 0.8 mm nozzle was determined to be the minimum based on the ability to print and thread the 1k CF prepreg. By this, the study converges to a setting for an increased fiber volume fraction $\left(V_{f}\right)$ in the printed composites.

Parametric optimization was studied to analyze the change in print performance with varied print parameters ($D_{n},\;l_{s}$ and ${\dot{V}}_{r}$). Idealistically, increased fiber content improves the mechanical properties of the print. Nevertheless, the prepreg tow needed a minimum amount of resin as a matrix material for a consistent and reliable printing process. So, the study converged to the maximum fiber content recipe with consistent printing performance and then investigated the mechanical properties offered by that recipe.

The $D_{n}\;$ also limited the $l_{s}$ values of the print. Intuitively, the printed raster’s centerlines should be spaced at a distance equal to the $D_{n}$. However, as the resin was dispensed at a liquid state around the prepreg, the printed line’s top surface produced an arched profile. This produced a waviness at the top surface of the printed layer. This waviness compounded with each subsequent layer and created void zones.

The ${\dot{V}}_{r}\;$ through the nozzle was a dependent yet important process parameter. The rate of resin required was dependent on the layer thickness, raster spacing, and the speed of printing. Depending on the other parameters, the ${\dot{V}}_{r}$ was adjusted to a rate so that the resin only exits through the outlet tip of the dispensing nozzle, and there was no resin backflow through the resin entry port. This set the maximum ${\dot{V}}_{r}$ for a $D_{n}.$ At the same time, ${\dot{V}}_{r}$ should be sufficient to satisfactorily limit the surface roughness of the printed layer, setting a minimum threshold value of ${\dot{V}}_{r}\;$ for each $D_{n}\;\&\;l_{s}$ settings. This is worth noting that this study focused on the parameter adjustments at the straight sections of the print lines. At locations where the printing track made a sharp turn, a different set of parametric adjustments was required. This is due to the rigidity of the CF tows and limitations involving cornering radius. This could be observed from the more uneven sections at the ends of the printed specimens in Figure 6. As the print parameters were only optimized for the straight sections, the corners suffered from unevenness, increased thickness, and surface abrasion. The surface unevenness could be minimized by layer-wise and dynamic adjustments of process parameters [48,50,65].

**Figure 7:**
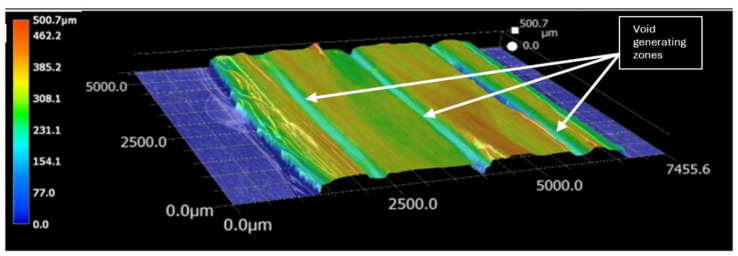
Topographic analysis of the printed surface.

The effects of the variables $D_{n},\;l_{s}$ and ${\dot{V}}_{r}$ on the average surface roughness $S_{a}$ were studied to find out the optimized ${\dot{V}}_{r}$. For better packing of the fiber tow in the composite with limited void zones, it was essential to study the topography of the printed surfaces. This was conducted by using a KEYENCE VHX digital microscope (Keyence Corporation of America, Itasca, IL, USA). Figure 7 shows the microscope-generated topographic profile of the top surface of a single-layer print.

Using each nozzle size, single layers of composite were printed with different ${\dot{V}}_{r}$ and $l_{s}\;$ settings. The $S_{a}\;$ measurements were taken in 10 mm × 10 mm sections from the middle part of printed layers. The most significant source of the $S_{a}\;$ changes was the valley between the two adjacent print lines because of the insufficient resin in that region. As layer thickness was kept fixed, the valley’s depth could be reduced by either increasing the ${\dot{V}}_{r}\;$ or by decreasing the spacing between the adjacent print lines.

The individual dimensions varied significantly for the peaks and valleys, even in the surfaces printed with the fixed printing parameters. A significant reason behind this variability in the surface finish was that the deposition of solid and liquid materials at the print had inherent positional uncertainty. Moreover, the shrinkage of the liquid resin during solidification was somewhat indeterminate. $S_{a}\;$ values over an area A were calculated using Equation (1). In this equation, z(x,y) was the vertical distance of the surface profile at any point (x,y). The z distances were measured from the average surface profile. Average surface profiles were calculated through the least square method.

(1) $$S_{a}=\frac{1}{A}\iint |z(x,y)|dxdy$$

The surface topography study was used to determine the resin flow rates in the test matrix. These three nozzle diameters were tested for the minimum and maximum spacings. After setting up the spacing boundaries, intermediate spacing configurations were tested for print performance. The test matrix is shown in Table 2. Composite specimens printed with the parameter configuration shown in Table 2 were tested for compositional analysis and mechanical characterization.

### 2.5. Testing

#### 2.5.1. Topographic Analysis

To quantitatively rank the quality of printed layers, the surface roughness $(S_{a})\;$ was measured using different parameter configurations. $S_{a}\;$ was tested under 40× enlarged microscopic imaging. To filter out the noise and general waviness of the build platform, the cutoff wavelength of roughness measurement was set from 100 µm to 2.5 mm. The fluctuations outside this wavelength range were filtered out. $S_{a}\;$ was calculated using Equation (1) Details about the topographical study are discussed in Section 3.1.

#### 2.5.2. Differential Scanning Calorimetry (DSC)

The DSC test was conducted with 3D printed composites and unreacted resin systems to ensure sufficient cure of the specimens after the post-curing stage. The amount of cure in the final product was investigated by comparing the heat flow curves of uncured and cured samples. These tests were conducted using a TA modulated differential scanning calorimeter (DSC Q1000, TA Instruments, New Castle, DE, USA). The temperature range for DSC tests was 40 °C to 200 °C. Heat flow was measured at a temperature ramp set at 10 °C/min. Standard aluminum pans were used for testing the printed specimens, and hermetic aluminum pans were used for testing the unreacted resin mixtures.

#### 2.5.3. Thermogravimetric Analysis (TGA)

3D printed specimens of neat resin (without fiber reinforcement) were thermogravimetrically analyzed to measure the ash content left by the matrix after burn-off. This ash residue amount was used to accurately calculate the composites’ $V_{f}$ from the burn-off test. The TGA test was carried out according to the ASTM E1131 standard [66]. TGA tests were conducted using TGA Q500 (TA Instruments, Series Q500, Eden Prairie, MA, USA). In the TGA tests, the specimens were heated from room temperature to 565 °C with a ramp rate of 10 °C/min. The mass differences between before and after heating were utilized to calculate the ash residue of the matrix materials.

#### 2.5.4. Micro-CT Testing

To measure the void content inside the composites, 20 (±0.5) mm × 18 (±1) mm × 2.4 (±0.2) mm printed coupons were CT scanned. This test was carried out using GE micro-CT equipment (model: v|tome|x s, General Electronics, Boston, MA, USA).

#### 2.5.5. Burn-Off Testing

Burn-off tests were conducted to determine the $V_{f}\;$ of the printed composites. Small sections (20 (±0.5) mm × 18 (±1) mm × 2.4 (±0.2) mm) of the printed composites were cut out and placed in the Lucifer Furnace (Model, RD4-KHE24, Warrington, PA, USA) to burn off the matrix materials. The burn-off tests were performed in reference to the ASTM D3171 standard [67]. The composite was heated at 565 °C for 6 h in a nitrogen environment. The weight loss in the burn-off test was used to calculate the $V_{f}\;$ of the printed composites.

#### 2.5.6. Tensile Testing

The tensile properties of the 3D printed composites were tested by an Instron load frame (Model 5567, Norwood, MA, USA). Glass fiber tabs were attached at the ends of the 3D printed composite specimens using two-part epoxy. All tensile tests were conducted according to the ASTM D3039 standard [68]. The tensile test specimens were 100 mm long and had a cross-sectional area of 18 ± 1 mm wide and 2.4 ± 0.2 mm thick. The gauge length used was 100 mm. Tensile loads were measured using a 30 kN load cell. The tensile strain and deformation were measured using a 25.4 mm extensometer.

#### 2.5.7. Flexural Testing

The printed composites’ flexural properties were tested by conducting a 3-point bending test using an Instron load frame (Model 5567, Norwood, MA, USA). The flexural tests were continued to specimen failure, and tests were set up according to the ASTM D7264 standard [69]. Flexural specimens had a cross-sectional area of 18 ± 1 mm wide and 2.4 ± 0.2 mm thick. The specimens were 100 mm in length. Flexural tests were performed with a 30 kN load cell at an Instron load frame (Instron, Series: 5567, Norwood, MA, USA). Complying with the standard, the span to thickness ratio of the specimen under flexural load was set at 32:1.

## 3. Results and Discussion

### 3.1. Surface Characteristics

**Figure 8:**
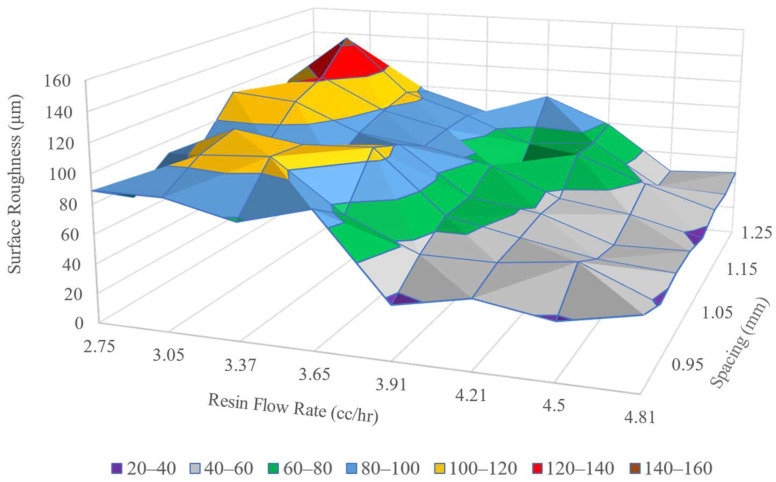
Surface roughness of single layers printed with 1.2 mm nozzles.

The surface roughness measured provided quantitative means for a print parameter configuration to be assessed, whether the configuration passed or failed for further specimen printing. Specimens printed with configurations that showed $S_{a}$ lower than the set threshold were taken for mechanical characterization tests. The maximum threshold value for $S_{a}\;$ was selected to be 60 µm. Figure 8 shows the plot of $S_{a}$ values obtained from surfaces printed with 1.2 mm nozzles.

Despite the fluctuations, the $S_{a}$ values of composite surfaces, printed with 1.2 mm nozzles, exhibited a trend toward achieving a minimum value below 50 µm. From Figure 8, it is observed that surfaces printed with $D_{n}=\;$ 1.2 mm and ${\dot{V}}_{r}\;$ > 4.21 cc/h configurations had $S_{a}$ values below the maximum threshold limit of 60 µm. These $l_{s\;}$ and ${\dot{V}}_{r}\;$ configurations are presented as the grey zones in the surface plot in Figure 8. To obtain maximum $V_{f}$, a specific $l_{s\;}$, ${\dot{V}}_{r}$ was chosen at the minimum value from where $S_{a}$ was consistently below the set threshold. For example, *S_a_* values are consistently under 60 µm for resin ${\dot{V}}_{r}$ ≥ 3.91 cc/h. when $l_{s\;}=$ 1 mm is used. So, to achieve maximum $V_{f}$, 3.91 cc/h. resin flow rate was used for $l_{s\;}=$ 1 mm with a 1.2 mm nozzle. For any spacing (with a 1.2 mm nozzle) the ${\dot{V}}_{r}$ was taken from the interfacial line between the grey and yellow regions of the surface plot in Figure 8.

**Figure 9:**
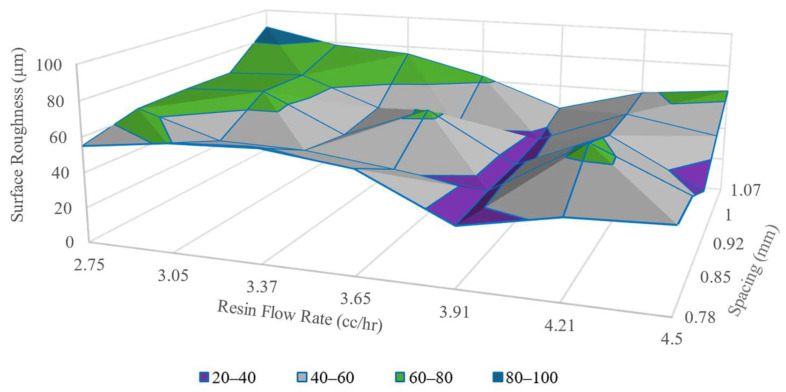
Surface roughness map for single layer prints with 1.00 mm nozzles.

**Figure 10:**
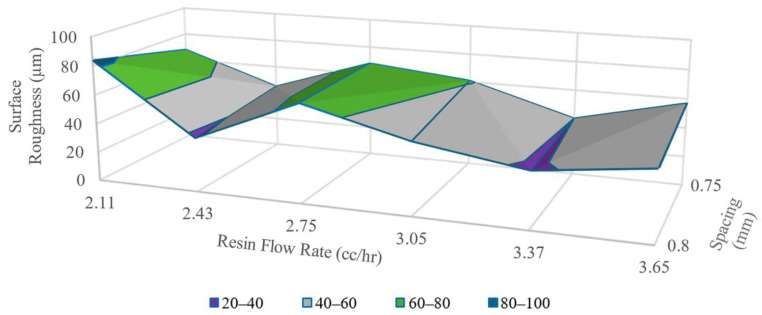
Surface roughness map of prints with 0.8 mm nozzles.

While $S_{a}$ varied significantly for configurations with 1.2 mm printing nozzles, $S_{a}$ for 1 mm and 0.8 mm nozzles fluctuated within a narrower range of values. This difference in extreme limits of $S_{a}$ values was observed because of the selection of tighter line spacings for smaller-sized nozzles. These selections of print settings left even slimmer space for the resin to occupy between the lines when small nozzle sizes were utilized. Figure 9 and Figure 10 show the $S_{a}$ maps for surfaces printed with 1 mm and 0.8 mm nozzles, respectively.

From Figure 9, it was observed that for ${\dot{V}}_{r}$ > 2.43 cc/h, the surface roughness starts to increase again. This observation indicated that ${\dot{V}}_{r}$ had critical points for each nozzle size and spacing configuration. When ${\dot{V}}_{r}$ was set above this critical value, resin started to overflow around the nozzle outlet lips, thus increasing the roughness of the printed surface. This observation also indicated that the minimum surface roughness achieved by this 3D printing setup was ~30–40 µm. This trend is previously reported in the literature [48].

In Figure 10, it can be noticed that, in contrast to other nozzle sizes, only two spacing settings were tested for surface topography analysis. The reason behind this was that the reduction of $l_{s\;}$ any lower than 0.75 mm resulted in constant failure during the print due to interference and the prepregs inability to make tight corners with such a small radius.

The results from the topographic analysis plots revealed the optimum resin flow rates for each nozzle diameter, spacing, and print speed configuration. Setting the optimum resin flow rate for printing the composite minimized the large void formation and print defects. These optimum resin flow rates were then set for 3D printing the composite specimens for compositional and mechanical characterization.

### 3.2. Curing

**Figure 11:**
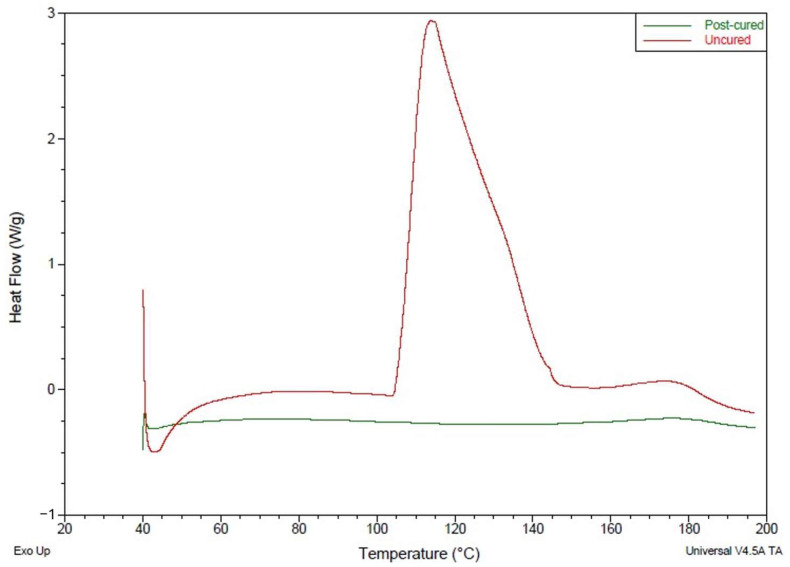
Heat flow curves of unreacted resins and post-cured composites during DSC tests.

For the practical applications of the additively manufactured composites, the matrix materials should be fully cured after manufacturing. In the manufacturing process of this study, the matrix material undergoes three stages of curing: partial curing during the prepregging, UV curing during the printing process, and, lastly, thermal curing at 130 °C. The final degree of cure was ensured by DSC results. As the resin goes through an exothermic chemical reaction during the curing stage, no exothermic heat flow should be observed from a satisfactorily cured matrix at elevated temperatures. The heat flow plots for the post-cured and uncured matrix in Figure 11 show that post-cured exhibited no exothermic heat flow at a temperature range of 30–200 °C. The uncured resin’s DSC plot showed a clear exothermic heat flow within this temperature range. This comparison (Figure 11) ensured the sufficient curing of 3D printed composite specimens after the post-curing stage.

### 3.3. Composite Composition

**Figure 12:**
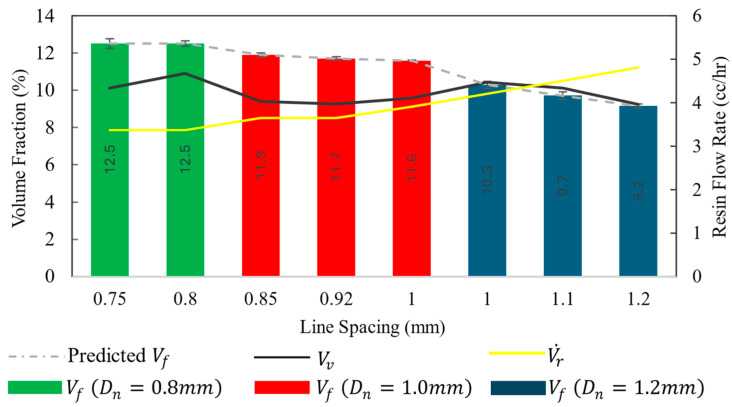
Composition of 3D printed composites with optimum resin flow rates.

Material constituent percentages were measured using a combination of micro-CT, TGA, and burn-off tests. The results of the micro-CT scan provided the void content, and TGA provided the ash residue of the matrix. Combining these results with the burn-off test result, the $V_{f}$ was calculated. The constituent composition of composites printed with different print parameter configurations is shown in Figure 12. The ${\dot{V}}_{r}$ for each configuration was determined from the lowest surface roughness settings measured by the topographic analysis. Higher ${\dot{V}}_{r}$ was required for larger $l_{s}\;$ configurations to maintain a $S_{a}\;$ at the print surface. The larger line spacing configurations thus produced composites with a slightly lower $V_{f}$. The differences of $V_{f}$ among different configurations were statistically significant with a *p*-value of $1.91\times 10^{-101}$. In contrast to $V_{f}$, the variation in void percentage among different configurations was statistically non-significant. Void percentage varied between a narrow range of 9.2 and 10.1%. This void percentage is higher than that of the composites manufactured in traditional methods. This high void percentage is a limiting factor for maximizing the composites’ mechanical performances. The resin shrinkage, trapped air, and layer inconsistencies are major reasons influencing the high void content in the printed composites. These factors could be limited by the incorporation of adaptive parametric adjustment methods in the 3D printing process [48]. This method requires in situ surface analysis equipment that feeds the printer controller information about real-time parameter adjustments [50]. These methods could also utilize machine learning algorithms for the detection of void-forming surface inconsistencies [65].

**Figure 13:**
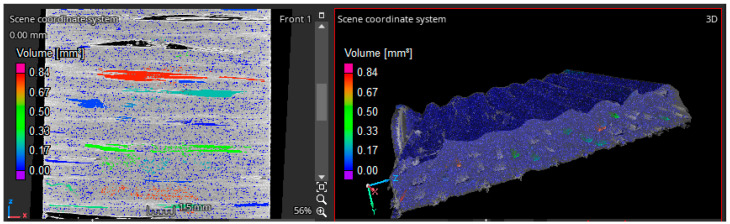
Micro-CT of 3D printed composite.

The constituents’ composition of the printed composites can be predicted from the print speed and material delivery rate. However, the liquid resin shrinkages at different curing stages produce internal voids. Voids were formed within the print lines and within the print layers even for an optimized resin flow rate based on the surface profiles. Rahman et al. studied this effect with a variable resin flow rate [48]. Figure 13 showed a similar void distribution in the micro-CT results. The dominant void distribution was aligned in the inter-line and inter-layer regions in the parallel direction to the print lines. So, for a constant resin flow rate 3D printing system, the composition predictive model should take the resin shrinkage effect to accurately predict the composition of the printed composites. Rahman et al. proposed such a predictive model based on an experimentally obtained shrinkage factor [64]. The model assumes that the internal voids in the composites are formed by resin shrinkage. In the current study, Rahman’s model is used in modified form to accommodate for the resin infusion of the prepregging stage. A shrinkage factor of k = 0.87 was used for the predictive model. The model is described in Equations (2)–(4), where print speed $S_{p}$ = 200 mm/min, density of CF $\rho_{cf}=1.76\;g/cc,$ density of resin $\rho_{r}=1.12$ g/cc, the fiber volume fraction of prepreg tow $V_{fp}$ = 0.67, weight of unit length of CF tow $w_{cf}^{\prime }=0.0685\;g/m$, weight of unit length of prepreg $w_{p}^{\prime }=0.0905\;g/m$, and fiber mass fraction of prepreg $m_{fp}=0.24$. The plot of the predicted $V_{f}$ in Figure 12 shows that the model worked accurately for this 3D printing system. This model can work as a tool for designing 3D printed composite components.

(2) $$V_{f}=\frac{\frac{w_{cf}^{\prime }S_{p}}{1000\rho_{cf}}}{\frac{w_{cf}^{\prime }S_{p}}{1000\rho_{cf}}+\frac{{\dot{V}}_{r}}{60}+\left(1-m_{fp}\right)\frac{S_{p}w_{p}^{\prime }}{\rho_{r}}}$$

(3) $$V_{m}=\frac{\frac{{\dot{V}}_{r}}{60}k}{\frac{w_{cf}^{\prime }S_{p}}{1000\rho_{cf}}+\frac{{\dot{V}}_{r}}{60}+\left(1-m_{fp}\right)\frac{S_{p}w_{p}^{\prime }}{\rho_{r}}}$$

(4) $$V_{v}=\frac{(1-k)\frac{{\dot{V}}_{r}}{60}}{\frac{w_{cf}^{\prime }S_{p}}{1000\rho_{cf}}+\frac{{\dot{V}}_{r}}{60}+\left(1-m_{fp}\right)\frac{S_{p}w_{p}^{\prime }}{\rho_{r}}}$$

### 3.4. Tensile Properties

**Figure 14:**
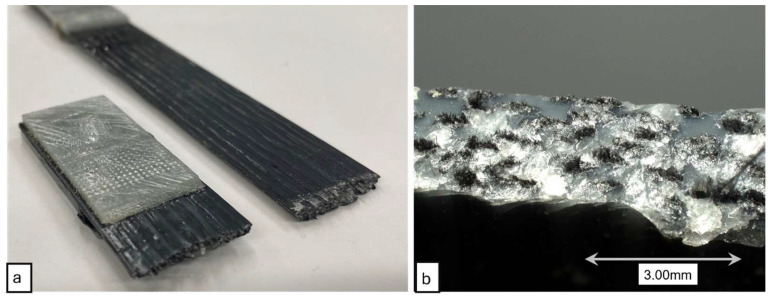
(**a**) Failed specimen under tensile loading. (**b**) Microscopic view of failure surface.

**Figure 15:**
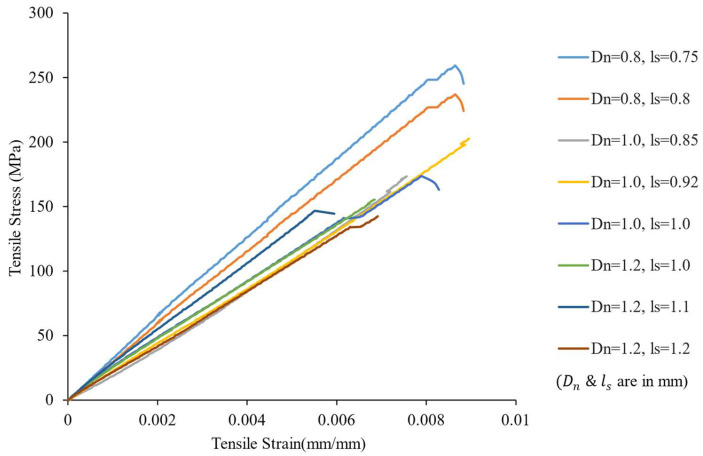
Stress-strain curves of composite specimens under tensile loading.

The 3D printed composite specimens were tested under tensile loading up to failure. The tensile specimens produced clean brittle failure modes, as expected from a CF composite utilizing a brittle matrix. Figure 14 shows the failure surface of the 3D printed composites under tensile loading. The fracture surfaces exhibited brittle failure of the composites. Fiber pullout was minimum, which indicated that the composites had good fiber wet-out and good fiber-matrix interfacial bonding. For each configuration of print parameters, five specimens were tested for tensile property determination. The average tensile strengths obtained from tensile tests are presented in Figure 15. In addition to experimentally obtaining the tensile properties, the tensile strength and modulus were theoretically calculated using the rule of mixtures (ROM). ROM for tensile strength and modulus are expressed by the Equations (5) and (6), respectively. In these equations $\sigma_{cu}$, $\sigma_{fu},$ and $\sigma_{mu}$ are ultimate strengths of composite, fiber, and matrix, respectively. Here, *E_c_*, *E_f_*, and *E_m_* are tensile elastic moduli of composite, fiber, and matrix, respectively.

(5) $$\sigma_{cu}=\sigma_{fu}V_{f}{+\sigma }_{mu}(1-V_{f}-V_{v})$$

(6) $$E_{c}=E_{f}V_{f}{+E}_{m}(1-V_{f}-V_{v})$$

The tensile stress-strain plots for composite specimens printed with different $D_{n}\;\&\;l_{s}$ are demonstrated in Figure 14. From this plot, it can be observed that tensile strength, modulus, and ultimate strain varied with varied $D_{n}\;\&\;l_{s}$. This plot represents sample specimen responses under tensile load. The average tensile behaviors of the composite specimens are discussed in the later sections.

**Figure 16:**
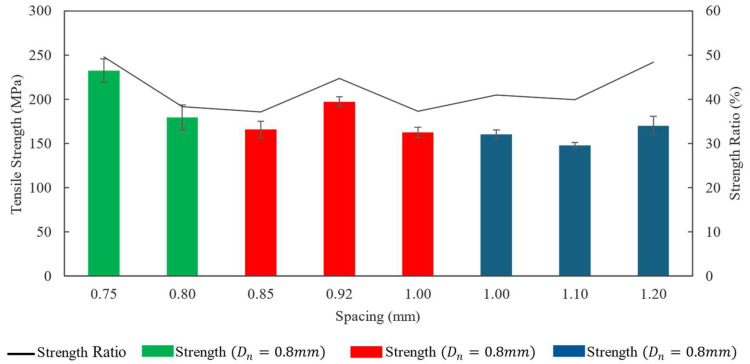
Tensile strength of 3D printed composites.

The specimens with the lowest spacing between the printed lines (0.75 mm) demonstrated the maximum average ultimate tensile strength of 232.35 MPa. A comparison of data points from Figure 15 showed that the $D_{n}\;$ and $l_{s}\;$ directly influenced both the $V_{f}$ and tensile properties of the composites. While keeping the ${\dot{V}}_{r}$ constant, increasing the spacing also increased the void content in the composite. This hike in void contents was observed in the plot in Figure 16 ($D_{n}=0.8\;mm)$. As a result, the hike in void percentage in the specimens printed with $D_{n}=$ 0.8 mm and $l_{s}=$ 0.8 mm reduced the ultimate tensile strength sustained by the composite specimens.

Figure 16 also plots the ratio of experimental to theoretical (from ROM calculations) tensile strength (strength ratio). This plot shows that the strength values obtained from the tensile test ranged between 37 and 50% of ROM predicted strength values. The fluctuation of the strength ratio could be a result of multiple factors, such as void fraction $V_{v}$, void distribution, fiber alignment, and statistical errors. The strength variation among the specimens of different print settings was statistically significant with a *p*-value < 0.0001. However, a comparison of the strength ratio within the results obtained from a fixed nozzle size indicates that the strength ratio is sensitive to void fractions. A probable cause for this sensitivity of strength ratio is the void distribution. The ROM prediction of tensile strength factored in the total void volume, but the concentration of void contents in close vicinity could further adversely affect the actual tensile strength. Thus, actual tensile strength dropped significantly with a slight increment of $V_{v}$, compared to ROM predicted values.

A 37–50% strength ratio is indicative of shortcomings in efficient load transfer between fiber and matrix. This study utilized a CF tow with a sizing optimized for an epoxy-based matrix, while the matrix was an acrylate polymer. This mismatch of interface compatibility could have contributed to the modest strength ratio. Furthermore, in the intra-tow regions, there was a significant amount of filament–filament contact instead of filament–matrix interface. These filament–filament interfaces do not contribute to the load transfer between the fiber and the matrix. Studies using Karam’s modified ROM model reported such an effect for 3D printed composites [36].

**Figure 17:**
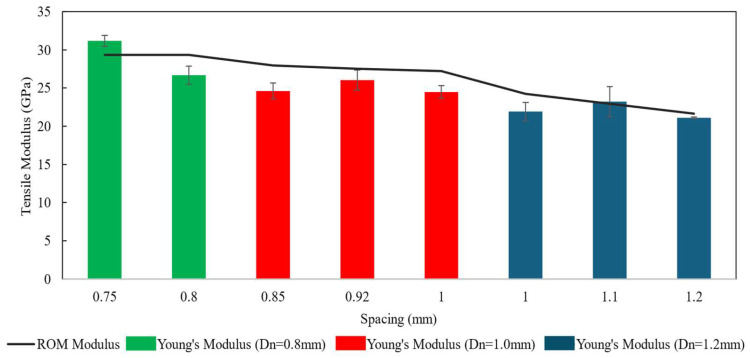
Young’s modulus of 3D printed composites.

The tensile strain at failure varied within a narrow range of 0.007 to 0.008 with statistically non-significant variation (*p*-value = 0.05). This failure strain is much smaller than the ultimate strain of both CF and the matrix. The significant void percentage in the composite could lead to this type of failure by the conjoining of crack networks originating from these voids. In contrast to strength and strain, the tensile modulus of the 3D printed composites exhibited good conformity with the ROM-predicted modulus. The variation in the tensile modulus of the printed composites produced from different print configurations was also statistically significant (*p*-value < 0.001). The tight placement of the print lines with narrow nozzle diameters, printed composites with a higher Young’s modulus. The trend and conformity of modulus are profoundly important. By utilizing the capability of altering the modulus of the 3D printed composites, it is possible to manufacture composite objects with a locally varying Young’s modulus within the object. This can enable the manufacturing of composites with engineered shapes under deformation. The average tensile moduli of printed composite specimens are plotted in Figure 17.

### 3.5. Flexural Properties

**Figure 18:**
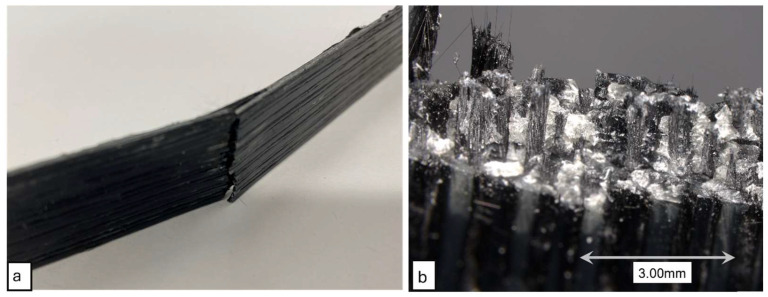
(**a**) Failure under flexural load. (**b**) Failure surface.

Flexural tests were performed to fracture the composite specimen under the point bending loads. Almost all test specimens’ failure mode was a bottom-face tensile failure with no visible signs of crushing at the top face. Figure 18 shows the fracture surface of a sample flexural test specimen. Microscopic investigation showed good fiber-matrix adhesion and delamination near the neutral plane.

**Figure 19:**
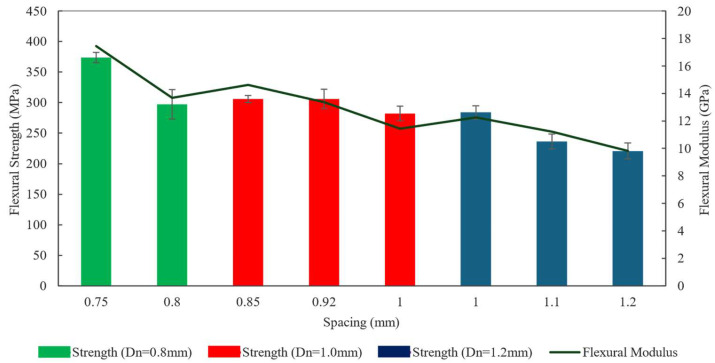
Flexural strength and modulus.

The flexural strengths of the printed composites are plotted in Figure 19. This plot revealed that the change in flexural strength followed almost the same trend as the change in $V_{f}$. The flexural modulus also followed the same trend. Deviation from this trend (prints with $D_{n}=$ 0.8 and $l_{s}=$ 0.8 mm) could be correlated to increased void content in the printed specimens. Specimens printed with the smallest $l_{s}\;$ exhibited the maximum average flexural strength of 373 MPa. Though $V_{f}$ was the same for the two spacing settings for 0.8 mm nozzles, the flexural strength of the prints with $l_{s}=$ 0.8 mm was significantly lower than that of $l_{s}=$ 0.75 mm. This reduction in flexural strength can be correlated to the increased $V_{v}$ of the prints with 0.75 mm spacing. However, compared to tensile and flexural results, the flexural strength showed a better match with the $V_{f}$ trend. This suggested that the flexural strength is comparatively less susceptible to the void fraction. This effect was probably observed since flexural failure takes place under the loading nose of the three-point bending test. Thus, it is less likely to be affected by a localized conglomeration of voids.

## 4. Conclusions and Future Recommendations

This study investigated the effect of process parameters on the mechanical properties of 3D printed continuous fiber-reinforced thermoset composites. By changing the dispensing nozzle diameter and spacing between the print lines, the composition of the 3D printed composite objects was altered. For each configuration of nozzle size and spacing, the material flow rate was optimized by topographic analysis of the printed composite surfaces. Mechanical characterization showed that the manipulation of variation in composition via parameter adjustments can lead to controlled changes in the mechanical properties of the 3D printed composites. The tensile strength and Young’s modulus were varied within the ranges of 148–232 MPa and 21.1–31.2 GPa, respectively. The flexural strength and modulus were varied within the ranges of 220.2–345.7 MPa and 10.2–16.5 GPa, respectively. These mechanical property alterations followed close, predictable trendlines.

3D printed continuous fiber-reinforced composites showed susceptibility to small changes in void content. Tensile and flexural strain at failure showed insignificant change due to composition alteration via parameter adjustment. In contrast, the tensile and flexural moduli exhibited trends that were in good agreement with the compositional trend induced by print parameter adjustments. This agreement underscores the 3D printing technique’s capability to engineer the mechanical properties of the printed composites. This technique possesses great potential for locally adjusting the elastic properties of composites. Local adjustment of elastic properties can be a useful tool for designing and engineering failure zones and deformation shapes of composite components.

Further detailed study is necessary to develop methods for better controlling the properties of 3D printed composites. 3D printed composites’ high void content limited their strength. Further studies aiming to control and reduce the void content and its distribution can produce high-performing 3D printed composites. In future studies, high-performing matrix material could be used. A stronger and tougher resin system, in conjunction with CF coated with optimized sizing for the resin system, could manufacture 3D printed composites with great mechanical strength and stiffness.

## Figures and Tables

**Table 1:** Material Properties.

Property	Toray 1K CF	Peopoly Deft Resin
**Tensile strength (MPa)**	3530	35
**Tensile modulus (GPa)**	230	0.75
**Elongation (%)**	1.5	6
**Density (gm/cm^3^)**	1.76	1.14
**Viscosity (cps at 25 °C)**	-	105

**Table 2:** Test matrix for the processing parameters.

	$D_{n}=$ 1.2 mm	$D_{n}=$ 1.0 mm	$D_{n}=$ 0.8 mm
Max spacing	$l_{s}\;$ = 1.2 mm ${\dot{V}}_{r}\;\;$ = 4.81 cc/h	$l_{s}$ = 1.0 mm ${\dot{V}}_{r}\;$ = 3.95 cc/h	$l_{s}$ = 0.8 mm ${\dot{V}}_{r}$ = 3.37 cc/h
Intermediate spacing	$l_{s}$ = 1.1 mm ${\dot{V}}_{r}\;$ = 4.5 cc/h	$l_{s}$ = 0.92 mm ${\dot{V}}_{r}\;$ = 3.62 cc/h	X
Minimum spacing	$l_{s}$ = 1.0 mm ${\dot{V}}_{r}\;$ = 4.29 cc/h	$l_{s}$ = 0.85 mm ${\dot{V}}_{r}\;$ = 3.62 cc/h	$l_{s}$ = 0.75 mm ${\dot{V}}_{r}\;$= 3.37 cc/h

## Data Availability

The data presented in this study are available on request from the corresponding author.
